# Transplantation of adipose-derived mesenchymal stem cells attenuates pulmonary fibrosis of silicosis via anti-inflammatory and anti-apoptosis effects in rats

**DOI:** 10.1186/s13287-018-0846-9

**Published:** 2018-04-19

**Authors:** Shangya Chen, Guanqun Cui, Cheng Peng, Martin F. Lavin, Xiaoying Sun, Enguo Zhang, Ye Yang, Yingjun Guan, Zhongjun Du, Hua Shao

**Affiliations:** 1 Department of Toxicology, Shandong Academy of Occupational Health and Occupational Medicine, Shandong Academy of Medical Sciences, Jinan, Shandong People’s Republic of China; 2 Department of Respiratory Medicine, Qilu Children’s Hospital of Shandong University, Jinan, Shandong People’s Republic of China; 3 Queensland Alliance for Environmental Health Sciences (QAEHS), the University of Queensland, Brisbane, QLD Australia; 4 University of Queensland Centre for Clinical Research (UQCCR), the University of Queensland, Herston, Brisbane, QLD Australia; 5 School of Medicine and Life Sciences, University of Jinan–Shandong Academy of Medical Sciences, Jinan, Shandong People’s Republic of China; 6 Department of Dermatology, Yueyang Hospital of Integrated Traditional Chinese and Western Medicine, Shanghai University of Traditional Chinese Medicine, Shanghai, People’s Republic of China

**Keywords:** Adipose-derived mesenchymal stem cells, Silicosis, Pulmonary fibrosis, Animal model, Transplantation, Cell therapy

## Abstract

**Background:**

Silicosis has been topping the list of high-incidence occupational diseases in developing countries and cannot be completely cured. Recent advances in stem cell research have made possible the treatment of various diseases including lung fibrosis. The application of stem cell therapy in occupational diseases, in particular the use of adipose-derived mesenchymal stem cells (AD-MSCs) in treatment of silicosis, has not yet been reported. The aim of the study is to explore the intervening effect of silica-induced lung fibrosis in rats.

**Methods:**

In this study, we investigated the anti-pulmonary fibrosis effects of the transplantation of AD-MSCs in rats in which lung fibrosis was induced by oral tracheal intubation with silica suspension. Twenty rats were divided into four groups: control group (*n* = 5), exposure group (*n* = 5), vehicle group (*n* = 5) and treatment group (*n* = 5). AD-MSCs were given to rats after exposure to silica for 24 h. Twenty-eight days after AD-MSC transplantation, we examined the organ coefficient, inflammatory cytokines, apoptosis, pathological and fibrotic changes in lung tissue.

**Results:**

Results showed that exposure to silica for 28 days induced an increase of the lung coefficient with significant pulmonary fibrosis. Treatment with AD-MSC transplantation led to a remissive effect on pulmonary fibrosis. We found that after AD-MSC transplantation the inflammatory response decreased and Caspase-3 protein expression significantly decreased with a significant increase of the Bcl-2/Bax ratio.

**Conclusions:**

Anti-inflammatory and anti-apoptosis of AD-MSCs may play important roles in their anti-pulmonary fibrosis effect. Our data suggest that transplantation of AD-MSCs holds promise for potential interference in the formation of silicosis through regulating inflammatory and apoptotic processes.

## Background

Silicosis refers to long-term inhalation and retention of silica dust in the lungs, mainly due to occupational exposure. The silicosis process involves the formation of silicon nodules, diffusion of pulmonary fibrosis and resultant diffused fibrotic pneumoconiosis [1]. Pathological characteristics of silicosis include alveolar epithelial cell injury and the formation of pulmonary fibrosis, but the mechanisms of silicosis are not fully clear. It is believed that inflammatory-related and apoptosis-related mechanisms play important roles in lung injury induced by silica dust and intervening/therapeutic routes targeting these pathways have been explored. For example, extensive attempts have been made to promote the treatment of silicosis through repairing injured cells but limited effects have been obtained. The use of stem cells as transplant tissue shows particular promise since stem cells have shown potency to repair damaged pulmonary tissue by replacing the endogenous damaged cells through cell regeneration and changing the microenvironment [2]. Recently, much progress has been made in understanding the mechanisms of mesenchymal stem cells (MSCs) in the aspects of regulating immunity and tissue remodeling in animal models of lung fibrosis [3]. In lung tissue rescue and repair, adult MSCs have been proposed to be a valuable therapeutic option due to their availability, immunomodulatory effects and anti-apoptotic and anti-inflammatory properties [4].

As mesodermal-derived cells, bone marrow-derived mesenchymal stem cells (BM-MSCs) are primarily obtained through bone marrow aspiration. Mature adipose tissue collection can help extract primary AD-MSCs to obtain autologous AD-MSCs. Later, these mesodermal-derived cells were proved to be pluripotent stem cells with multilineage differentiation potential and horizontal differentiation ability [5]. AD-MSCs have similar biological characteristics, immunosuppressive effects and multilineage differentiation potential to BM-MSCs which can differentiate into adipocytes, osteocytes, chondrocytes, myocytes and neural precursor cells [6–8]. However, because of the low content of BM-MSCs in bone marrow, prolonged time and complicated procedures for cell collection, culture and purification are required before transplantation. In contrast, AD-MSCs are plentiful in adipose tissue which is easy to access. To obtain the same amount of MSCs through bone marrow aspiration, the donor may only need to provide a smaller amount of adipose tissue, with relatively less pain and feasibility. These factors ensure AD-MSCs have a wider range of applications in stem cell treatment.

In recent years, a number of studies have tried to use MSCs for treatment of pulmonary injury. BM-MSCs transfected with HGF have been reported to be effective in improving pulmonary fibrosis in patients with silicosis. Researchers transfused autologous BM-MSCs-HGF into silicosis patients, which reduced pulmonary small nodules significantly, with pulmonary function and inflammation of patients gradually ameliorated [9]. MSCs-HGF may be a potential treatment option for pulmonary injury. AD-MSC transplantation was also found to promote angiogenesis in the injured lung tissue by enhancing the expression of HGF [10]. The mechanism of action of MSCs in pulmonary diseases is thought to include regulating inflammation and promoting angiogenesis [11–14], and is affected by the quality of MSCs used [15–18]. AD-MSCs have been shown to be effective in repairing and regenerating lung tissue [19–21], including ameliorating idiopathic pulmonary fibrosis [22]. A recent study found that AD-MSCs can mitigate bleomycin-induced lung fibrosis in aged mice [23]. However, silicosis-induced pulmonary fibrosis is different from bleomycin-induced pulmonary fibrosis in etiology. So far, there are currently no reported studies on AD-MSC treatment of silica-induced pulmonary fibrosis. Therefore, the aim of this study is to explore the preventive effect and mechanism of AD-MSC treatment for silicosis. To this end, we cultivated AD-MSCs for transplantation in rats with oral tracheal intubation with silica suspension and evaluated organ coefficients, pathological and fibrotic changes in lung tissue and inflammatory cytokines in lung tissue, and measured expression of the protein involved in the mitochondria-associated apoptotic pathway. Data from the study will provide evidence for the effects of AD-MSCs on silica-induced fibrosis and helpful information in the application of AD-MSCs for therapeutic purposes in the future.

## Methods

### Experimental animals and experimental design

Male Sprague Dawley (SD) rats weighting 180–200 g were purchased from Beijing Vital River Laboratory Animal Technology Co. Ltd (certificate No. SCXK (Jing) 2012–0001) and maintained under standard housing conditions (temperature 18–24 °C; relative humidity 45–60%; light and dark cycle 12 h:12 h). Food and water were provided ad libitum. All animals were treated according to the protocols evaluated and approved by the experimental animal ethical committee of Shandong Academy of Medical Sciences.

**Fig. 1:**
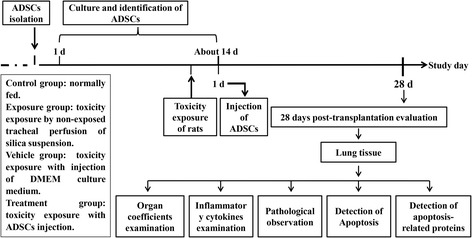
Animal experiment design for anti-apoptotic effects of transplantation of AD-MSCs in adult rats. ADSC adipose-derived stem cell, d days, DMEM Dulbecco’s modified Eagle’s medium

Animal treatment and experimental design are shown in Fig. 1. Twenty adult male SD rats were randomly divided into four groups: control group (*n* = 5), which were normally fed; exposure group (*n* = 5), which were exposed to silica; vehicle group, (*n* = 5), in which DMEM culture medium was administered by intravenous injection 24 h after silica exposure; and treatment group (*n* = 5), which received 5 × 10^5^ AD-MSCs (suspended in DMEM culture medium) by intravenous injection 24 h after exposure to silica. On the 28th day after transplantation, the animals from each group were evaluated for different parameters (Fig. 1).

### Culture and identification of stem cells

AD-MSCs from adipose tissue of 4-week-old healthy male adult SD rats (*n* = 3) were isolated and cultured according to a previous report with slight modifications [24]. Briefly, the SD rats were sacrificed by 3% pentobarbital sodium anesthetic overdose. After being placed in 75% ethanol for about 5 min, rats were dissected on a super-clean bench. The abdominal skin was cut along the midline of the abdomen until adipose tissue of the groin was exposed. Adipose tissue of the groin was collected and washed three times with phosphate buffered solution (PBS) containing 100 IU/ml penicillin and 100 μg/ml streptomycin (ThermoFisher Scientific, USA). After being transferred into a dry Petri dish, the tissue was cut quickly by sterilized ophthalmic scissors for 10 min. The tissue pieces were collected and digested with collagenase dissolved in Dulbecco’s modified Eagle’s medium (DMEM) (with 5% BSA, 5 mg/ml) (ThermoFisher Scientific, USA) at 37 °C for 60 min. After centrifugation at 1000 rpm (Sorvall ST8R; ThermoFisher Scientific, USA), the cell pellet was suspended in complete medium (DMEM with 10% embryonic stem cell-qualified fetal bovine serum, 100 IU/ml penicillin and 100 μg/ml streptomycin) (Biological Industries, USA). The cell suspension was filtered through a cell filter with 70-μm pores. A cell suspension with 10^4^ cells/ml was obtained and incubated in CO_2_ incubator (Labserv CO-150; ThermoFisher Scientific, USA) after being washed with DMEM and centrifuged as already described. The cells were observed daily and photographs were taken under inverted microscope (IX70; Olympus, Japan).

AD-MSCs were identified and selected by flow cytometry (FCM) with CD44, CD45, CD90, CD73 and CD11b antibodies. After being subcultured to the third generation, AD-MSCs at 80% confluence were washed twice with PBS followed by digestion with 0.25% trypsin–EDTA (ThermoFisher Scientific, USA). The cells were then centrifuged at 1000 rpm and washed with PBS. After incubation with antibodies and their isotype controls (1:100) (Becton Dickinson and Co., USA) at 4 °C for 30 min, the cells were flowed through the cytometer at about 1000 cells per second. Results of FCM were analyzed by FlowJo software (FlowJo, LLC, USA).

AD-MSCs were also identified by adipogenic, osteogenic and chondrogenic differentiation. After being subcultured to the third generation, AD-MSCs at 80% confluence were induced in adipogenesis, osteogenic and chondrogenic differentiation complete medium (using adipogenesis, osteogenic and chondrogenic differentiation kits from ThermoFisher Scientific, USA). After induction for 21 days, cells were fixed with 4% paraformaldehyde for about 20 min. The intracellular lipids accumulated in the induced AD-MSCs in adipogenic cultures were stained with Oil Red O (Sigma-Aldrich, USA). The mineralized osteogenic cultures were stained with Alizarin Red S (Sigma-Aldrich, USA) to detect calcium deposition. The mucopolysaccharide accumulated in chondrogenic cultures were stained with Toluidine Blue (Sigma-Aldrich, USA). Photographs were taken under the inverted microscope.

### Silica-induced silicosis in rats

We improved the method of oral tracheal intubation with silica suspension to induce silicosis in rats. The micron-sized silica (approximately 80% between 1 and 5 μm; Sigma-Aldrich, USA) was autoclaved and made into a suspension at 50 mg/ml in normal saline. Rats were fixed on the operating table after 3% pentobarbital sodium anesthesia. Incisors of rat were pulled down by surgical suture and the tongue of the rat was pulled out by sterile forceps with the light of exceeded luminosity lamp-house toward the neck of the rat. The glottis was the bright spot opening and closing with breath which could be seen through the mouth. Then we wiped the mucus near the glottis with a cotton swab. When the glottis was open, the indwelling venous needle was inserted into the trachea. Silica suspension (1 ml) was injected quickly into the cannula. Then the rat was gently shaken for about 5 min to distribute the suspension uniformly in the lungs. The rats in the control group were perfused with 1.0 ml of sterile normal saline.

### Transplantation procedure

AD-MSCs were resuspended at a concentration of 5 × 10^5^ cells/ml for transplantation which was performed on rats exposed to silica for 24 h. The cell dosage was 1 × 10^6^ cells/kg. Briefly, rats were anesthetized with 3% pentobarbital sodium. The rat tail was sterilized by 75% ethanol. Then 1 ml of cell suspension with 5 × 10^5^ cells/ml was injected over 2 min using a disposable vein infusion needle which was attached to a disposable syringe of 1 ml. In order to minimize cell leakage, the needle was left in place for 2 min after injection before withdrawal. The vehicle group had DMEM culture medium “transplanted” in the same way.

### Inflammatory cytokine measurement

TNF-α, IL-1β, IL-6 and IL-10 were detected by ELISA using different ELISA kits (ThermoFisher Scientific, USA) according to the manufacturer’s protocols. Briefly, rats from each group were euthanized 28 days post transplantation. The lungs of rats were rapidly removed, weighed and frozen at −80 °C for further experiment. After being diluted, detection A solution was added into the plate at 100 μl per well and placed at 37 °C for 1 h. After the plate was washed three times, detection B solution was added as detection A and placed at 37 °C for 30 min. The plate was then washed five times, and 3,3′,5,5′-tetramethylbenzidine (TMB) was added at 90 μl per well and placed at 37 °C for 20 min. Finally, sulfuric acid was added at 50 μl per well as the termination solution. The optical density (OD) of each well of a 96-well plate at 450 nm was analyzed using a microplate reader (SpectraMax 190; Molecular Devices, USA).

### Histopathological and fibrotic examination

Rats were deeply anesthetized with 3% pentobarbital sodium 28 days post transplantation. The body weight was measured and the heart, liver, spleen and lungs of rats were isolated and excised with a razor blade and weighed, and the organ coefficient (the ratio of the organ weight to the body weight) was calculated. The organ coefficient is an important indicator to evaluate toxic effects of test substances in toxicology research. Then, the lungs of rats were collected and some parts were fixed in 4% (v/v) paraformaldehyde. After embedding in paraffin blocks, tissues were sectioned into 5 μm slice, and then mounted onto glass slides. Tissues on the glass slides were stained with hematoxylin and eosin (H&E) for histopathological evaluation, and stained with Masson’s trichrome stain for fibrotic examination. Photographs were taken using an optical microscope (BX51; Olympus, Japan). The slides were examined by pathologists who were blinded to the identity and analysis of pathology sections. The results were peer-reviewed by other certified veterinary pathologists. Fibrotic changes were quantified by modified Ashcroft score with a grade of 0–8 to represent alveolar structure from normal lung to total fibrous obliteration of lung fields [25].

### Apoptosis detection

The lungs of rats were processed as already described for terminal deoxynucleotide transferase (TdT)-mediated dUTP nick end labeling (TUNEL) using an in-situ cell death detection kit (Roche, Switzerland) according to the manufacturer’s instructions. Briefly, lung slides of each group were fixed with 4% paraformaldehyde in 0.1 M phosphate buffer (pH 7.4). After incubation with proteinase K (100 μg/ml), sections were washed with PBS, and permeabilized with 0.1% Triton X-100. The sections were then washed with PBS twice and incubated in TUNEL reaction mixture. After sections were rinsed again, the converter-POD was used to visualize sections with 0.02% 3,3′-diaminobenzidine (DAB). After counter-staining with Mayer’s hematoxylin, sections were mounted on the slides which were gelatin-coated and placed at room temperature overnight to be air-dried. Positive results were brown-stained nucleus. An apoptotic index was evaluated to analyze differences between groups in apoptosis detection. The apoptosis index was determined from 10 blindly selected high-power fields for each slice by counting the number of positive cells in 500–1000 cells per field to calculate the percentage of positive cells.

### Western blot assay

We used western blot analysis to detect the expression of Bax, Bcl-2 and Caspase-3 proteins in lung tissue of rats from each group. The lungs were processed as already described and lung tissues were homogenized for protein extraction. After being resuspended in homogenization buffer, the tissues were centrifuged for 10 min (12,000 rpm, 4 °C). Supernatants were collected as protein suspension. SDS-PAGE (12% resolving gels at 120 V, 5% stacking gels at 75 V) was used to separate protein samples. Then protein samples were transferred to PVDF membranes (200 mA, 1 h). The membranes were blocked with 5% nonfat dry milk followed by incubation with different primary antibodies and β-actin (Santa Cruz, USA) overnight at 4 °C. After being washed with TBST, the membranes were incubated with secondary antibodies (Santa Cruz, USA) at 1:1000 dilutions for 1.5 h at room temperature. After being washed with TBST, the membranes were treated with enhanced chemiluminescence (ECL detection kit; Pierce) and exposed to the Odyssey CLx near-infrared fluorescence imaging system (LI-COR, USA). Results were quantified by AlphaEaseFC software (Alpha Innotech, USA). We performed experiments in triplicate to ensure reproducibility.

### Statistical analysis

Data are presented as means ± SD. Statistical analysis was performed using one-way analysis of variance (ANOVA). Data for each group were compared with other groups by least-significant difference (LSD) for significance. All statistical analyses used SPSS software (IBM SPSS Statistics 20.0, USA). Results were considered statistically significant at *p* < 0.05*.*

## Results

### Culture and identification of AD-MSCs

**Fig. 2:**
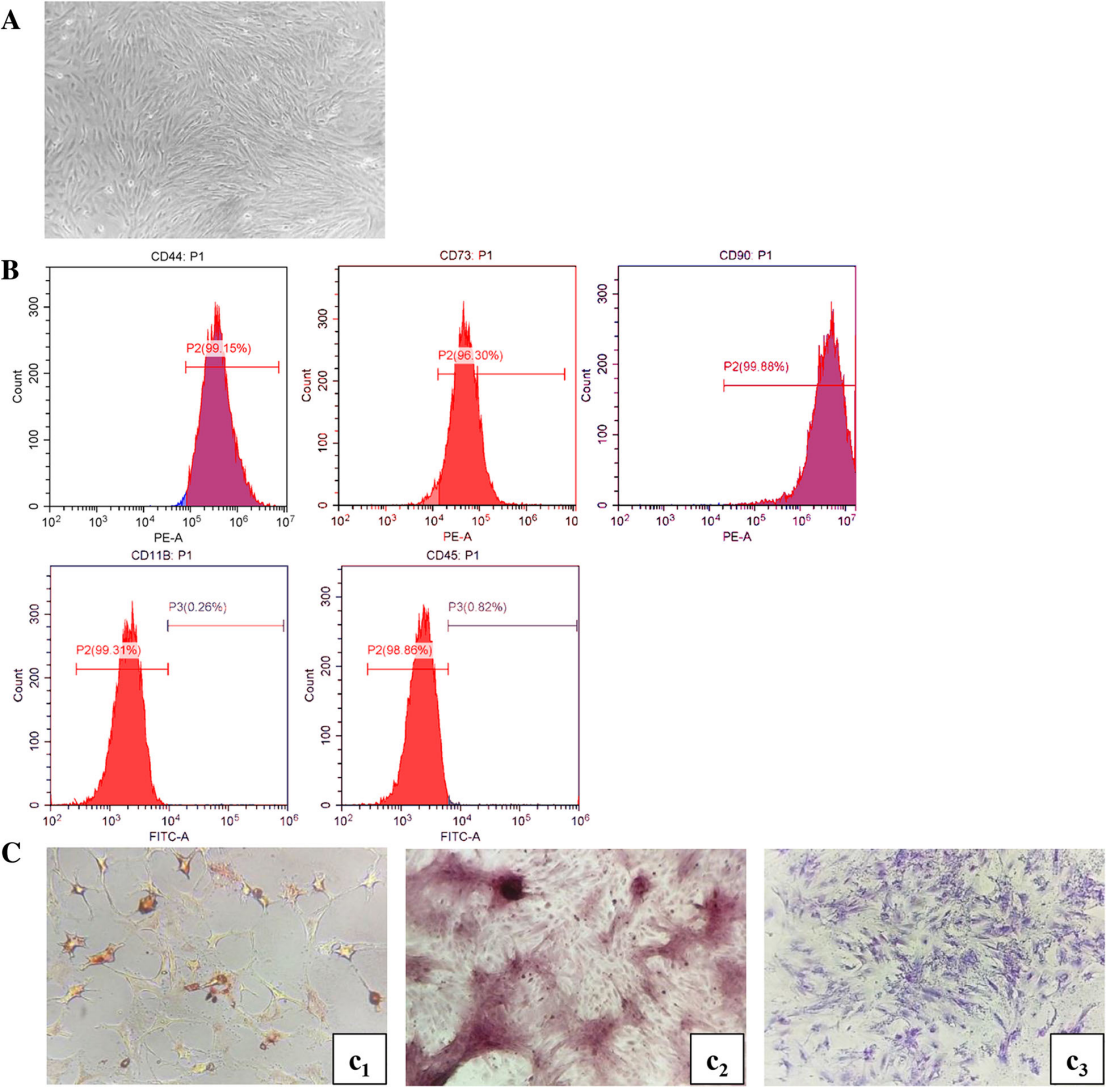
Isolation, cultivation and characterization of rat adipose-derived mesenchymal stem cells (AD-MSCs). **A** Morphology of AD-MSCs (rat AD-MSCs displayed spindle-shaped morphology, ×100). **B** Immunophenotype of AD-MSCs. Immunophenotype of rat AD-MSCs analyzed by flow cytometry. Most cells expressed CD44, CD73 and CD90, but were CD45-negative and CD11b-negative. **C** Multipotency of AD-MSCs. Rat AD-MSCs could differentiate into adipogenic (c_1_, ×200), osteogenic (c_2_, ×100) and chondrogenic (c_3_, ×200) lineages when cultured in differentiation medium. FITC fluorescein isothiocyanate, PE phycoerythrin

Primary cells were mainly spindle-shaped and partially polygonal, and showed rapid proliferation in the first 3 days. With the cell expansion progressed, most cells were long spindle-shaped and fibroblast-like with the number of hybrid cells decreased significantly, and the proliferation rate of AD-MSCs decreased gradually. After expansion to the third generation, the cells were long spindle-shaped and evenly distributed, and cells were whirlpool-like or chrysanthemum-like aggregated at a density of 100%. The cell morphology was more consistent with a faster growth rate. Flow cytometry measurement of cells at the third generation showed that expression of CD44, CD73 and CD90 was positive (99.15%, 96.30% and 99.88%), with negative expression of CD11b and CD45 (0.26% and 0.82%). Meanwhile, adipogenic, osteogenic and chondrogenic differentiation of cells at the third generation all succeeded. Thus, these results confirmed that the vast majority of cells in the third generation were AD-MSCs (Fig. 2).

### Organ coefficients of rats

The organ coefficient is considered a commonly used indicator of animal status. After rats were sacrificed, organs were removed and rinsed with normal saline. The organs were then blotted dry on filter paper and immediately weighed. The ratio of organ weight/body weight was considered the organ coefficient. There was no significant difference among the heart coefficient of rats in different groups. Compared to the control group, the lung coefficients of rats significantly increased in the treatment group, exposure group and vehicle group (*p* < 0.05) (Table 1).

**Table 1:** Organ coefficients of rats after surgery (mean ± SD, *n* = 5)

Group	Lung coefficient (%)	Heart coefficient (%)
Control group	0.57 ± 0.04	0.32 ± 0.05
Exposure group	0.90 ± 0.08^a^	0.31 ± 0.02
Vehicle group	0.89 ± 0.11^a^	0.30 ± 0.01
Treatment group	0.86 ± 0.24^a^	0.31 ± 0.02

^a^*p* < 0.05, vs control group

### AD-MSC transplantation recovers the silica-induced inflammatory cytokines

Inflammatory cytokines of lung tissue were evaluated after rats were sacrificed (Table 2). Compared to the control group, the TNF-α, IL-1β, IL-6 and IL-10 levels of rats significantly increased in rats in the exposure group and vehicle group (*p* < 0.05). There was no significant difference between the exposure and vehicle groups for each inflammatory cytokine. Notably, the TNF-α, IL-1β, IL-6 and IL-10 levels in the treatment group significantly decreased compared to the vehicle group (*p* < 0.05). The expression levels of TNF-α, IL-6 and IL-10 significantly decreased in the treatment group, with the IL-1β level decreased compared to the exposure group (*p < 0.05*). There was also a significant increase of IL-1β in rats from the treatment group compared to the control group.

**Table 2:** Inflammatory cytokines in lungs of rats after surgery (mean ± SD, *n* = 5)

Group	TNF-α (pg/ml)	IL-1β (pg/ml)	IL-6 (pg/ml)	IL-10 (pg/ml)
Control group	31.95 ± 8.06	443.18 ± 376.22	153.00 ± 106.11	165.63 ± 32.09
Exposure group	108.44 ± 16.90^a^	1355.97 ± 397.43^a^	521.30 ± 193.05^a^	334.45 ± 77.70^a^
Vehicle group	108.41 ± 15.10^a^	1600.92 ± 234.88^a^	569.65 ± 126.07^a^	389.74 ± 53.36^a^
Treatment group	43.07 ± 8.33^b,c^	1018.24 ± 102.88^a,c^	164.81 ± 51.66^b,c^	180.81 ± 38.44^b,c^

*IL* interleukin, TNF tumor necrosis factor

^a^*p* < 0.05*,* vs control group

^b^*p* < 0.05, vs exposure group

^c^*p <* 0.05, vs vehicle group

### AD-MSCs mitigate the lung pathology changes by silica

**Fig. 3:**
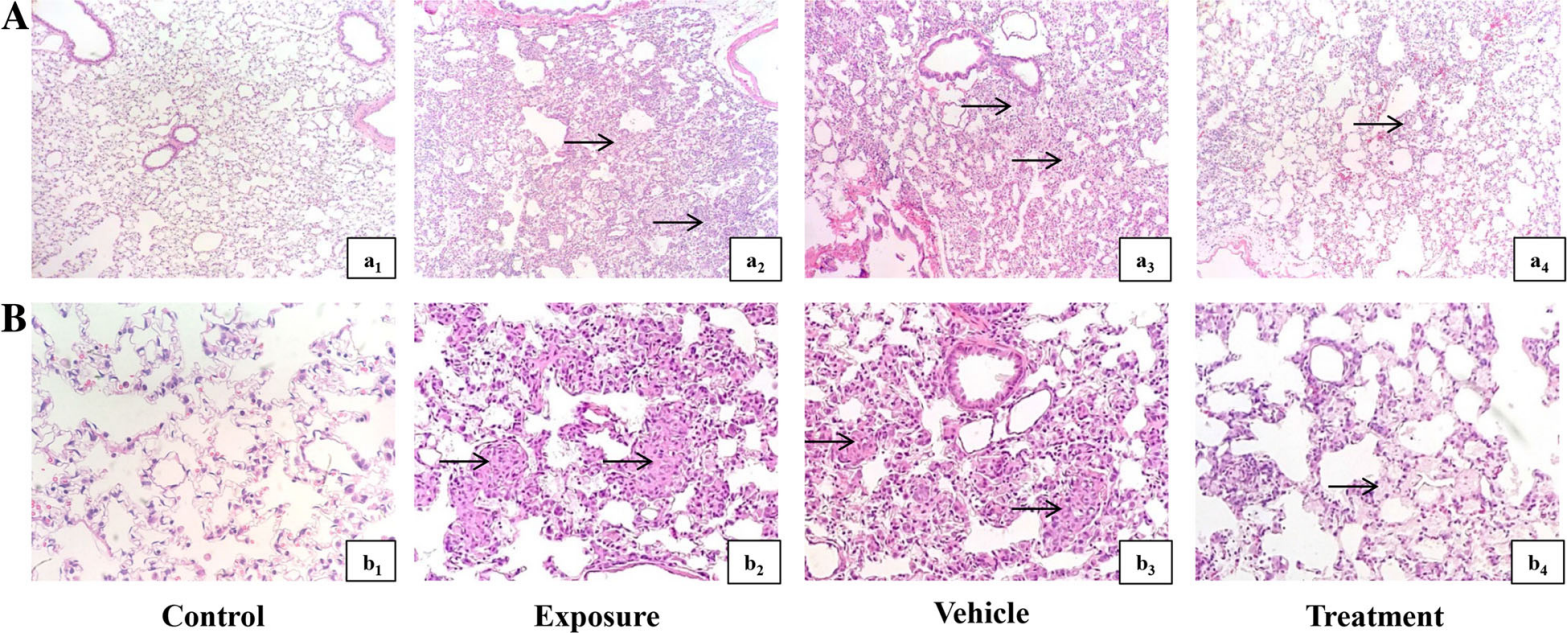
H&E staining of lung tissue in each group. **A** ×100; **B** ×400. (a_1_, b_1_) Control group, normal lung morphology: thin lined interalveolar septa with well-organized alveolar space. (a_2_, b_2_) Exposure group, distorted lung morphologies: collapsed alveolar space with inflammatory exudates, wider and thickened interalveolar septa, initial formation of silicon nodules. (a_3_, b_3_) Vehicle group, similar to exposure group. (a_4_, b_4_) Treatment group, ameliorative lung morphology: lessoned collapsed alveolar space with inflammatory exudate, less widened and thickened alveolar septum, reduced pronounced nodules of silicon. Arrows point out pathological changes of lung tissue

**Fig. 4:**
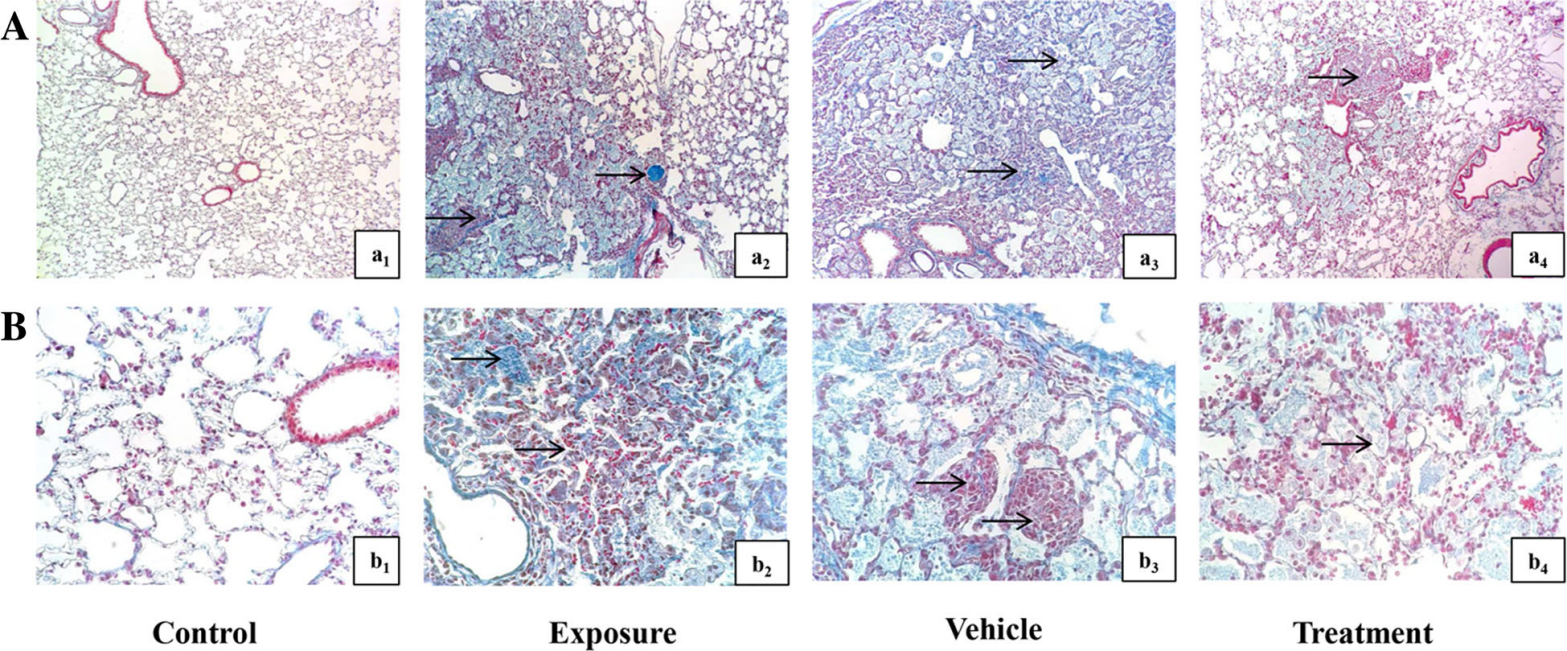
Masson staining of lung tissue in each group. **A** ×100; **B** ×400. (a_1_, b_1_) Control group, normal lung morphology: almost no deposition of collagen in lung parenchyma. (a_2_, b_2_) Exposure group, distorted lung morphologies: collagen fiber agglomerates, initial formation of silicon nodules and dense accumulation of collagen. (a_3_, b_3_) Vehicle group, similar to exposure group. (a_4_, b_4_) Treatment group, ameliorative lung morphology: lung sections showing reduced collagen deposition, less alveolar thickening and reduced amounts of collagen. Arrows point out pathological changes of lung tissue

**Fig. 5:**
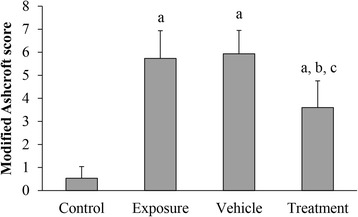
Severity of pulmonary fibrosis evaluated by modified Ashcroft score. Presented as mean ± SD (*n* = 5). ^a^*p* < 0.05, vs control group; ^b^*p* < 0.05 vs exposure group; ^c^*p* < 0.05 vs vehicle group

The alveolar structure of rats from the control group was intact with no inflammatory cell infiltration or fibrosis, as shown in Fig. 3Aa_1_, Bb_1_. Lung alveolar structures in rats from the exposure and vehicle groups were severely destroyed with a large amount of inflammatory cell infiltration, phagocytic cells and silicon nodules (Fig. 3Aa_2_, a_3_). Telangiectasia, interstitial lymphocytic infiltration, alveolar epithelium disarrangement and pulmonary vascular wall thickening were observed in rats from the exposure and vehicle groups (Fig. 3Bb_2_, b_3_). Alveolar structure destruction, scattered fusion of silicon nodules, inflammatory cells and lymphocytes were significantly reduced in the treatment group compared with the exposure group (Fig. 3Aa_4_, Bb_4_). Collagen fibers, mucus and cartilage were stained blue, while cytoplasm, muscle, cellulose and glial were stained red in Masson staining. After Masson staining, normal collagen fiber stent could be observed in the lung tissue of the control group (Fig. 4Aa_1_, Bb_1_). There was a large amount of collagen fibers deposited in the pulmonary mesenchyme, especially around bronchi and vessels in the exposure group and vehicle group (Fig. 4Aa_2_, a_3_, Bb_2_, b_3_). Collagen fiber deposition could also be found in the treatment group, but the area of deposition was decreased compared to the exposure group or vehicle group (Fig. 4Aa_4_, Bb_4_). The result of modified Ashcroft score evaluation was consistent with the result of the slice observation, as shown in Fig. 5.

### AD-MSCs reduced the silica-induced cellular apoptosis

**Fig. 6:**
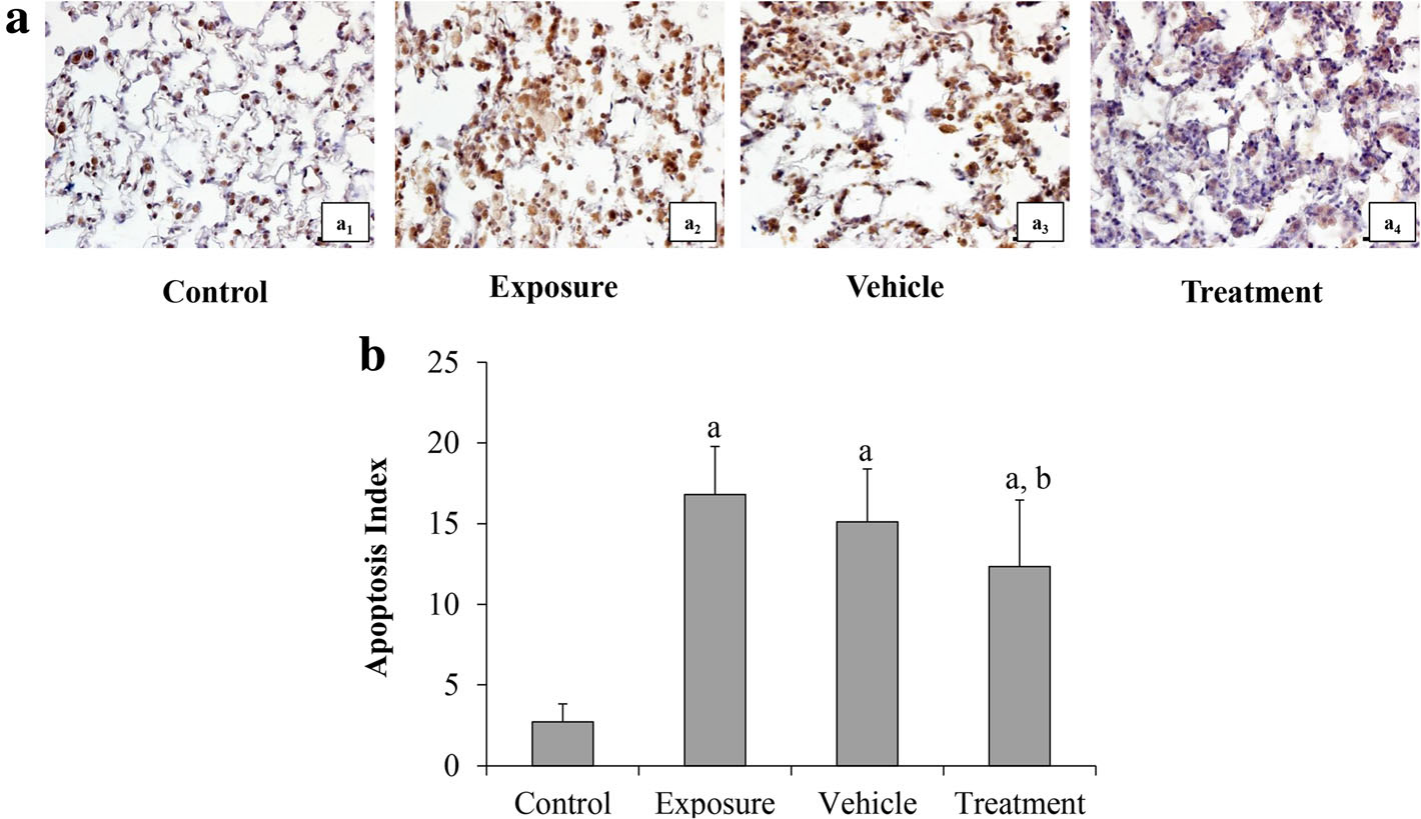
Cellular apoptosis of lung tissue in each group (TUNEL staining). **a** ×400: (a_1_) control group; (a_2_) exposure group; (a_3_) vehicle group; (a_4_) treatment group. **b** Apoptosis index of each group. ^a^*p* < 0.05 vs control group; ^b^*p* < 0.05 vs exposure group

We used the TUNEL method to detect the apoptosis level in lung tissues (Fig. 6). TUNEL-positive cells were observed to distribute in rats of the exposure, vehicle and treatment groups 28 days after transplantation. The apoptosis index showed much difference among the control group and the three other groups. An increased apoptosis index in the exposure, vehicle and treatment groups was observed compared with control group. The apoptosis index in the treatment group was significantly decreased compared with that in the exposure group (*p* < 0.05). No significant difference of the apoptosis index was seen between the vehicle group and the exposure group.

### Expressions of Bax, Bcl-2 and Caspase-3

**Fig. 7:**
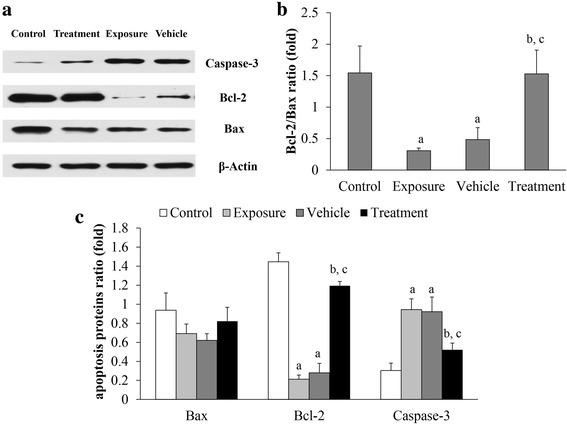
Expression levels of Bax, Bcl-2, Caspase-3 and β-actin proteins of lung tissue in rats 28 days after transplantation. **a** Western blot analysis of Bax, Bcl-2, Caspase-3 and β-actin proteins of lung tissue in rats. **b** Bcl-2/Bax ratio of lung tissue in each group. **c** Relative ratio of Bax, Bcl-2 and Caspase-3 protein of lung tissue in rats. ^a^*p* < 0.05 vs control group; ^b^*p* < 0.05 vs exposure group; ^c^*p* < 0.05 vs vehicle group

We found that silica exposure led to upregulation of Caspase-3 protein and downregulation of Bax and Bcl-2. After oral tracheal intubation with silica suspension, expression of Caspase-3 protein was significantly upregulated, while expression of Bax and Bcl-2 proteins was significantly downregulated (Fig. 7). In rats with AD-MSC transplantation, compared with those in the exposure and vehicle groups, Caspase-3 protein significantly decreased while Bax and Bcl-2 protein expression increased. Further analysis showed that the ratio of Bcl-2/Bax in the exposure and vehicle groups was significantly decreased, and the Bcl-2/Bax ratio in the treatment group was significantly increased when compared with the exposure group and vehicle group.

## Discussion

Recently, much attention has been paid to stem cell therapy in experimental silicosis since it appears to be susceptible to therapeutic intervention. Transplantation of BM-MSCs has been demonstrated to be a promising treatment for therapy of silicosis [26]. Clinical study has shown that transplantation of autologous BM-MSCs can effectively reduce pulmonary fibrosis with and improve lung function in patients with silicosis [24]. However, with current techniques, it is hard to obtain stem cells from bone marrow and this hinders the use of BM-MSCs. In contrast, AD-MSCs have a wider range of sources and are more readily available. In addition, liposuction is more likely to be accepted by patients compared with bone marrow puncture in clinical practice. To take these advantages, we selected AD-MSCs for intervening in experimental silicosis. The effect of AD-MSCs on silica-induced silicosis has not been reported. In this study, we successfully obtained AD-MSCs from adipose tissue of rats and cultured them in vitro. Primary AD-MSCs showed rapid proliferation in the first 3 days. After transfer to the third generation, the cells with a long spindle-shaped morphology were more uniform with a faster growth rate (Fig. 2). Then, we verified the cells by checking the expression of surface molecules and their adipogenic, osteogenic and chondrogenic differentiation ability (Fig. 2).

Pulmonary fibrosis is characterized by a certain degree of lung inflammation and abnormal tissue repair involving a complex network of cytokines. These events result in increased collagen gene expression and abnormal collagen deposition in the lungs that eventually produce fibroblast foci [27]. In order to observe the distribution of inflammatory cells and detect collagen deposition in lung interstitium, we used H&E staining and Masson staining to evaluate differences in the progression of pulmonary fibrosis between groups. Rats from the exposure groups showed positive staining, which suggested the formation of lung fibrosis (Figs. 3 and 4). The quantitative score of pathological analysis of pulmonary fibrosis was then conducted by estimation of the Modified Ashcroft scale [25]. We found that inflammatory cells aggregated and collagen was deposited in the lung, accompanied by severe distortion of pulmonary structure and large fibrous areas in rats exposed to silica suspension (Figs. 3 and 4). After AD-MSC transplantation, decreased inflammatory cells and collagen deposition in the lung tissue, reduced damage to the lung structure and formation of small fibrous masses were found in the lung interstitium (Fig. 4). The results showed that early intervention of AD-MSCs could reduce inflammatory reaction and slow down the process of pulmonary fibrosis.

Researchers hypothesized that the inflammatory reaction mechanisms underlying MSC therapy of pulmonary fibrosis were mediated by paracrine signaling (Reference). The therapeutic effect of BM-MSCs was found to trigger IL-1RA secretion to suppress the upregulated IL-1 and TNF-α proteins to protect the lung against damage and fibrosis [26]. Consistent with several previous studies using fetal membrane-derived stem cells and preconditioned BM-MSC transplantations as bleomycin-induced pulmonary fibrosis therapies [27, 28], we demonstrated that AD-MSC transplantation can reduce the pulmonary inflammatory response of rats after oral tracheal intubation with silica suspension, indicating the migration and homing of AD-MSCs toward the lungs through intravenous infusion to affect the inflammatory response to silica exposure. Downregulated expression of TNF-α, IL-1β, IL-6 and IL-10 proteins indicated that AD-MSCs can also protect the lungs from injury and fibrosis through an anti-inflammatory process, which was consistent with ample experimental evidence in MSC-treated pulmonary fibrosis [28–30]. Macrophages can stimulate epithelial cells by activating and releasing inflammatory cytokines TNF-α, IL-1, IL-6 and IL-10. Activated epithelial cells can regulate local immunity by recruiting locally inhabiting immunocompetent cells to provide intercellular and intracellular communication through autocrine and paracrine signaling pathways [31]. IL-1β and TNF-α mainly activate the nuclear factor-κB pathway [32, 33], while IL-6 and IL-10 exert their complex actions through multiple pathways like the Janus kinase 2/signal transducers and activators of transcription 3 pathway and the p38 mitogen-activated protein kinase/extracellular signal-regulated kinase pathway [34, 35]. TNF-α, IL-1β and IL-6 are regarded as proinflammatory cytokines, and IL-10 is generally considered to be an anti-inflammatory cytokine. It was also shown that overexpression of IL-10 aggravates silica-induced pulmonary inflammation and fibrosis in mice, which might relate to the promotion of Th2-type cytokine reaction and increased expression of IL-4 and IL-13 [32]. We found a downregulated expression of IL-10 at day 28 after transplantation, which might indicate the benefit of low expression of IL-10 in lung tissue during pulmonary fibrosis prohibition exerted by AD-MSC transplantation. In this regard, the effect of AD-MSC transplantation on pulmonary inflammatory factor release may directly alter the formation of pulmonary fibrosis through an anti-inflammatory pathway.

Apoptosis, inflammatory reaction and their cascade reactions can signify secondary damage after lung injury [36, 37]. Therefore, we tested the anti-apoptotic effect of AD-MSCs in a rat silicosis model and found that the apoptosis index of lung cells in AD-MSC-treated rats was decreased compared with that of the exposure group and vehicle group. We also found that the apoptosis of pulmonary cells in rats exposed to silica involved regulation processing of Bax, Bcl-2 and Caspase-3, thus probably resulting in the apoptotic death of cells in lung tissue. The increase in Caspase family protease activity is associated with apoptosis, in which Caspase-3 plays a pivotal part in the apoptosis of bleomycin-induced pulmonary fibrosis [38]. Further, our data showed decreased apoptosis in the treatment group using TUNEL assay (Fig. 6). Thus, the results suggested that AD-MSC transplantation in rats exposed to silica suspension exerted an anti-apoptotic effect.

Anti-apoptotic protein Bcl-2, pro-apoptotic protein Bax and Caspase-3 play an important role in the progression of apoptosis [39]. Bax regulates apoptosis by forming homologous dimers or heterodimers with Bcl-2 to produce an apoptotic regulatory system. Owing to the stability of heterodimer Bax–Bcl-2 compared to Bax–Bax, the ratio of Bcl-2/Bax regulates the occurrence of apoptosis and determines cell survival. Pulmonary cells are in a relatively stable proportion of pro-apoptotic protein Bax and anti-apoptotic protein Bcl-2 in physiological conditions, maintaining the steady-state balance of pulmonary structure and function. The Caspase family mainly exists in the form of zymogen in cells. When stimulated by the apoptotic signal, initiator Caspases are activated, and then the executioner Caspases. They induce apoptosis by decomposing substrate proteins. It has been shown that Caspase-3 may play an important role in apoptosis and has the effect of removing inhibition to mediate feedback amplification [40]. Therefore, we examined the expression of Bax, Bcl-2 and Caspase-3 in apoptosis by western blot assay in order to investigate the mechanism of apoptosis suppression after AD-MSC transplantation in rats with oral tracheal intubation with silica suspension. The results showed that the ratio of Bcl-2/Bax in the treatment group was significantly increased compared with the exposure group and vehicle group (Fig. 7). Although the expression levels of Bax and Bcl-2 proteins showed no significant difference among groups after exposure, we found significant downregulation of Caspase-3 in the treatment group. Therefore, we hypothesize that the high ratio of Bcl-2/Bax may be directly related to the release of Caspase-3, which eventually leads to apoptosis of lung tissue after exposure to silica. AD-MSC transplantation played an anti-apoptotic role in suppressing the downregulation of Bcl-2 protein in rats after oral tracheal intubation with silica suspension. Accordingly, the inhibition of Caspase-3 on account of the decrease in the ratio of Bax/Bcl-2 can result in declined apoptosis in the treatment group. These findings further confirmed that AD-MSCs appear to exert pulmonary protection and reduce the process of apoptosis by inhibiting the expression of proteins related to the mitochondrial apoptotic pathway in the secondary damage of experimental silicosis in rats. Thus, the data from this study indicated that AD-MSCs can mitigate silica-induced lung fibrosis in rats. We also realized that this is preliminary work from the point of view of translation study and more work is required to establish the intervening and even therapeutic effects of AD-MSCs in silica-induced lung fibrosis. A study is underway to explore the effects of AD-MSC on an established silicosis model and compare their efficacy with BM-MSCs.

## Conclusions

Transplanted AD-MSCs can significantly reduce the pulmonary inflammatory response induced by silica suspension in rats. AD-MSCs can also inhibit mitochondrial apoptosis-related protein expression, thereby inhibiting the process of silicosis. Although the apoptotic pathways require verification, we provide ample evidence for the further study of AD-MSCs in the treatment of silicosis. Our data suggest that AD-MSCs may have a beneficial effect on pulmonary fibrosis and may represent the basis of a new treatment for patients with silicosis. The successful development of stem cells in silicosis therapy will require a better understanding of host–graft interaction, the microenvironment and intrinsic characteristics, and subsequent greater functional improvement. Ongoing behavioral and biochemical assessment of long-term positive effects of AD-MSCs will provide further insight regarding the therapeutic potential of AD-MSCs for silicosis.

## Abbreviations

AD-MSC: Adipose-derived mesenchymal stem cell
ANOVA: One-way analysis of variance
BM-MSC: Bone marrow-derived mesenchymal stem cell
DMEM: Dulbecco’s modified Eagle’s medium
H&E: Hematoxylin and eosin
HGF: Hepatocyte growth factors
MSC: Mesenchymal stem cell
OD: Optical density
PBS: Phosphate buffered solution
SD: Sprague Dawley
TUNEL: Terminal deoxynucleotide transferase (TdT)-mediated dUTP nick end labeling
